# Effect of Geometric Error on Friction Behavior of Cylinder Seals

**DOI:** 10.3390/polym13193438

**Published:** 2021-10-07

**Authors:** Ange Lin, Jian Wu, Haohao Li, Zhe Li, Benlong Su, Youshan Wang

**Affiliations:** 1 Center for Rubber Composite Materials and Structures, Harbin Institute of Technology, Weihai 264209, China; 20S030009@stu.hit.edu.cn (A.L.); lizhe0223@sina.com (Z.L.); subenlong@hit.edu.cn (B.S.); wangys@hit.edu.cn (Y.W.); 2 Department of System Design, SVOLT Energy Technology Co., Ltd., Baoding 071000, China; lhh9403@163.com

**Keywords:** cylinder, dynamic friction, finite element simulation, geometric error, sealing ring

## Abstract

The tribological characteristics of the cylinder directly affect the operation accuracy of the pneumatic servo system. However, the geometric error has a significant effect on its tribological behavior and the related research is insufficient. Thus, the dynamic friction process of rubber seals has been investigated considering the influence of geometric errors. Firstly, based on the self-made friction test platform, the friction force of the rubber seals was studied and the influence law of geometric error on the contact area of the rubber seal ring was revealed. Secondly, the numerical model of the friction and contact of the rubber seals for the cylinder segment was developed by using the finite element simulation method and the influence laws of machining errors, such as roundness and straightness on the friction characteristics, were revealed. Finally, synergy effects of roundness and straightness in the friction behavior of rubber seals considering geometric errors was investigated, which lays a foundation for the accurate prediction of cylinder dynamic mechanical properties.

## 1. Introduction

The cylinder is one of the most common actuators in pneumatic servo control systems. The complex friction characteristics directly affect the performance of the system, especially in the occasions of high-precision positioning or low-speed motion trajectory tracking. Model-based friction compensation is the main way to overcome the adverse effects of friction on the system performance. Therefore, the development of an accurate model of cylinder friction is very important.

In recent years, more and more research studies have been carried out for studying the influence of cylinder surface roughness on the friction process. The effect of the surface texture and roughness of the softer and harder mating materials on the friction force during the sliding process was analyzed. Results indicate that the surface texture affects the friction under the sliding contact conditions [1]. The influence of waviness and roughness on cylinder liner friction behavior was also studied [2]. The friction performance of the diamond-like texture on the surface of the hydraulic cylinder was analyzed and optimized [3]. The surface roughness reduces the transition range of the boundary layer and significantly affects aerodynamics in the critical Reynolds number range [4]. Both experimental data and predictions show that when the relative roughness is less than 1%, the roughness has little effect on the flow characteristics. According to the standard, a new relationship between the predicted friction coefficient and the critical Reynolds number was also fitted [5]. In order to consider the influence of skewness, the Weibull distribution function was used to characterize the rough height distribution. Results show that the developed model can well predict the initial running-in behavior of the piston ring assembly/cylinder liner system under engine conditions [6]. The concept of disturbance error was introduced to describe the influence of accumulated errors in the manufacturing process. Additionally, geometric models have been developed to describe the relationship between geometric errors by using surface approximation technology [7].

The friction force of cylinders varies with time and there are serious unmodeled dynamics and unknown disturbances in the pneumatic system. A compensation friction model of the cylinder has also been developed. The steady-state friction characteristics of cylinders with good low-speed performance can be represented by ordinary friction models and the friction characteristics are mainly determined by the piston seals [8]. In order to study the effect factors in friction process, rather than the relative movement of the contact surface, the friction behavior of the hybrid pump control hydraulic system was studied. The pressure difference and acceleration terms were introduced into the LuGre friction model and the simulated friction force results of the updated LuGre model were compared with the measured experimental results to verify the new friction model [9]. A piston dynamics model considering surface roughness was established by Ricardo-Pisdyn software. Results show that appropriately increasing the depth of the cavity and optimizing the clearance can significantly reduce the cumulative wear load of the piston and improve both its friction and wear performance [10]. The relevance of friction in the main cylinder chamber was emphasized and the technology to compensate for its negative effects was discussed. Referring to the correctly identified linear model of the brake system, the pressure control system was designed [11]. An analytical friction model was developed to calculate the driving force, which is required to slide an object over a surface subject with coupling of longitudinal and lateral vibration. The subroutine of the friction model was developed, which can be integrated into contact simulation in Abaqus [12]. A numerical model of surface prediction for microfabrication was proposed. The error variable was applied to the proposed numerical model. The correctness of the numerical prediction model was verified by micro-cutting experiments [13]. An unknown friction using on/off reversing valve was used to position control of hydraulic cylinders [14]. Aimed at the arm electro-hydraulic system, a dynamic friction feedforward compensation method with the improved Stribeck model was designed. The results show that the proposed Stribeck model and friction compensation control method effectively eliminate the phenomenon of low-speed creep and amplitude flattening, and improve the control performance of the system [15]. A friction dynamics model was developed to estimate the minimum film profile, frictional force, and frictional power loss in the high-pressure zone of a high-performance engine. The Rk parameter was used to characterize the roughness of the lining to obtain a better surface representation [16]. Here, a strong friction compensation model was developed in the rodless cylinder system. The actuator saturation linear feedback control law was proposed to further improve the control performance [17]. The compensation model of the mechanical positioning unit was proposed and the T-SPL tool was used to realize the direct position measurement, which significantly reduced the position error caused by heat [18]. Three kinds of friction models, namely the steady-state friction model, LuGre model, and modified LuGre model, have been studied to analyze the influence on the accuracy of cylinder motion simulation. The RLG model can achieve the best consistency among the three friction models [19]. Numerical simulations clearly show the effects of elastoplastic deformation and friction. Results show that the established stiffness model provides a complete reference for solving surface contact problems [20]. The influence of the friction model on the simulation accuracy of hydraulic cylinders was studied. The new improved LuGre model can accurately predict the behavior of hydraulic cylinders [21]. An adaptive robust controller with dynamic friction compensation based on the LuGre model was constructed. It can guarantee the specified transient performance of motion tracking and the final tracking accuracy [22]. However, most existing model-based friction compensation schemes use simple classical models, which are not sufficient for applications with high precision position requirements.

The unreasonable geometric tolerance distribution of cylinders leads to the large fluctuation of the cylinder friction performance, which directly affects the friction performance of the sealing ring. Therefore, considering the influence of geometric errors in the inner wall of the cylinder on the friction characteristics of the seal ring, the dynamic friction of the cylinder seal was developed by the finite element method, which is basic for the dynamic performance of the cylinder. This research study has an important reference value to the design of cylinder geometry precision and seal structure.

## 2. Experiment Setup

### 2.1. Sealing Ring Friction

**Figure 1:**
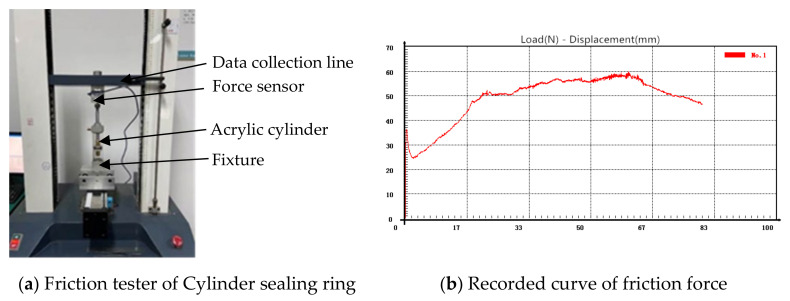
Principles of friction force testing for cylinder sealing ring.

**Figure 2:**
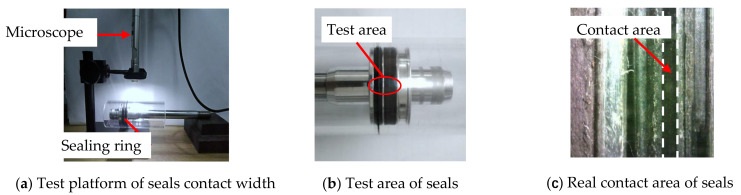
Measurement of contact width between seals and cylinder.

The MDBB40-10 cylinder was taken as the research object, while the equivalent transparent cylinder (made by polymethyl methacrylate) was combined with the original seals and pistons to form the friction pairs for studying the influence of geometric errors. Here, the material of the cylinder was polymethyl methacrylate (PMMA) and the seal ring material was nitrile butadiene rubber (NBR) in the friction testing. The friction test platform of the cylinder seal ring is shown in Figure 1, wherein the cylinder seal ring was driven by the servo motor to achieve uniform motion and then the dynamic friction force between the cylinder and the seal ring was tested (see Figure 2). In Figure 1, (a) is the cylinder seal ring friction test platform, and (b) is the real-time friction test data curve. In Figure 2, (a) is the test platform for the contact width of transparent cylinder, (b) is the test area for the contact width of transparent cylinder, and (c) is a partial enlarged view of the contact area.

For studying the influence of geometric errors, eight positions of 10 mm, 20 mm, 30 mm, 40 mm, 50 mm, 60 mm, 70 mm, and 80 mm from the top of the cylinder piston to the limit position of the bottom of the cylinder were taken as test points. Each point was tested three times with a test speed of 200 mm/min. In the test, the cylinder was set to move from a fixed test point and the next test was carried out back to the initial point after the movement. Then, the friction data obtained was recorded and processed.

### 2.2. Contact Width of the Sealing Ring

**Figure 3:**
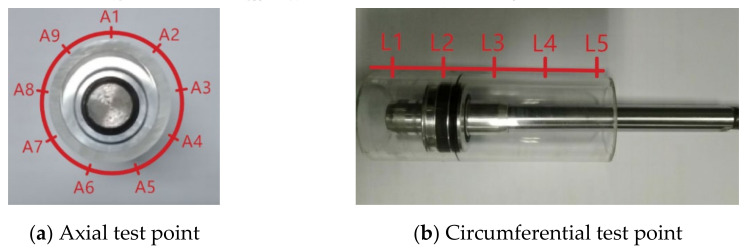
Distribution of test points for the cylinder contact width.

Due to the influence of roundness errors, the contact width between the seal ring and the inner wall of the cylinder in the circumferential direction varied, which in turn affected the friction. To study the difference in the contact width of each point in the circumferential direction at each axial position in the cylinder wall, an optical measurement platform based on the B011 digital microscope was developed. The test equipment and measurement method of the contact area are shown in Figure 2. In order to study the influence of roundness and straightness errors, several test points were selected for measurement in both the axial and circumferential directions. Five test points were selected in the axial direction (the total length of the axial test was 13mm and the interval of each point was 2.8 mm) and one test point was selected at an average interval of 40° in the circumferential direction; thus, there were 9 test points in the circumferential direction. The distribution of the test points is shown in Figure 3. Here, each test point was repeated 3 times and the data were recorded and processed. Finally, the average value was used in the study.

## 3. Finite Element Model

### 3.1. Constitutive Model

**Figure 4:**
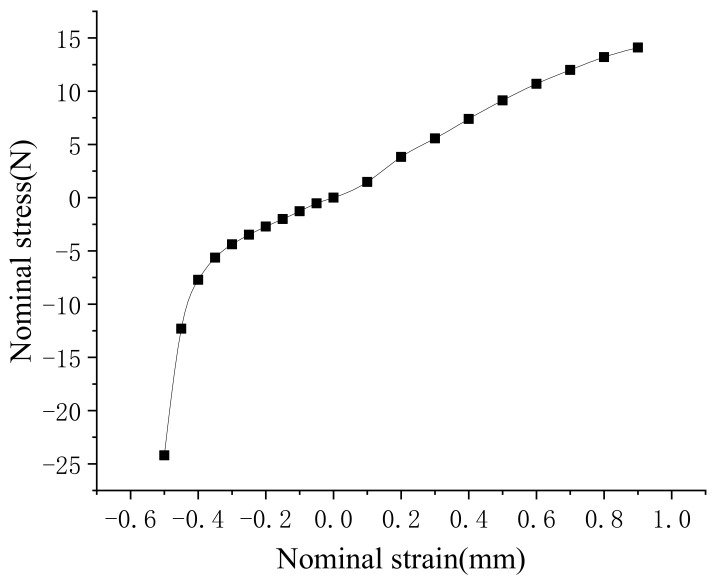
Stress–strain curve.

Based on the tensile test, the stress–strain relationship of the NBR material was obtained, which is shown in Figure 4. 

It can be seen that the NBR material had a strong non-linear behavior. The Yeoh model is simple and commonly used to fit the hyperelastic materials, such as rubber. A good fitting had been obtained based on the non-linear behavior of NBR, thus the Yeoh constitutive model was used in the finite element simulation.

The strain energy function W of the Yeoh model is given by:

(1) $$W_{Yeoh}=C_{10}\left(I_{C}-3\right)+C_{20}{\left(I_{C}-3\right)}^{2}+C_{30}{\left(I_{C}-3\right)}^{3}$$

where the *C*_10_, *C*_20_, and *C*_30_-yeoh model is the material coefficient.

Based on the test dates of the mechanical properties, the values of the material coefficients *C*_10_, *C*_20_, and *C*_30_ of the nitrile rubber material were fitted by the Abaqus software, which is shown in Table 1.

### 3.2. Meshing and Boundary

**Figure 5:**
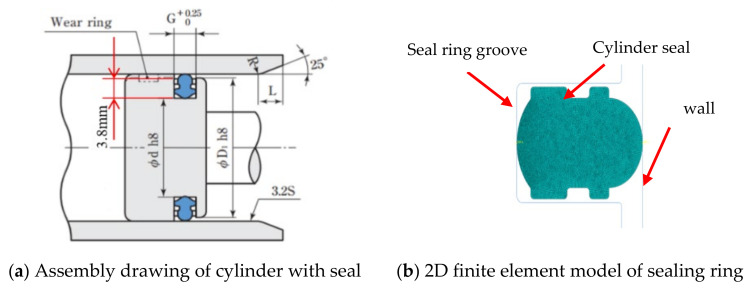
Finite element model of the sealing ring.

**Figure 6:**
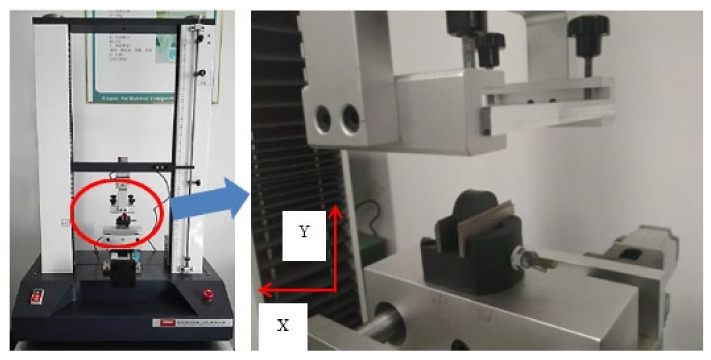
Friction tester.

Figure 5 shows the assembly size drawing (a) and meshing model of the cylinder seals (b). In this model, the mesh element type of the seal ring is C3D4H. The penalty model was used in the contact between the seal ring and the cylinder wall, and the friction coefficient was 0.1 under the condition of lubricating grease. Here, the friction coefficient between the seal ring and the cylinder wall (depicted in Figure 6) was measured by the friction tester (the test speed was 200 mm/min and the maximum contact pressure was about 2 Mpa). Here, the seal ring groove surface was fixed and then the cylinder wall surface was moved to the seal ring groove by applying the displacement load to the seal ring, with the compression amount of 0.5 + ΔR_Ai_ mm (0.5 is the nominal assembly compression amount, ΔR_Ai_ is the compression amount caused by the roundness error, etc.).

## 4. Results and Discussion

### 4.1. Contact Width

**Figure 7:**
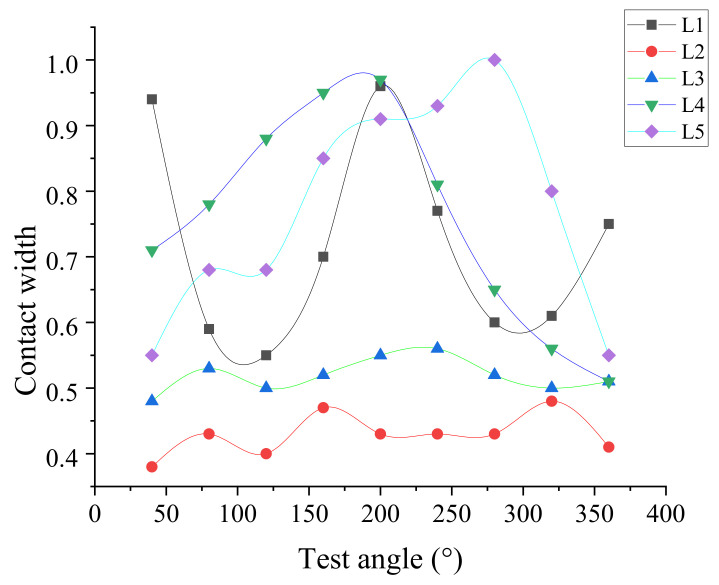
The contact width of each angle of the cylinder.

Figure 7 shows the contact width of the *A_i_* point in the circumferential direction of the cylinder seal ring in the axial direction *L_i_*.

Results indicate that the synergy effects of roundness and straightness have a significant influence on the contact width of the actual seal.

**Figure 8:**
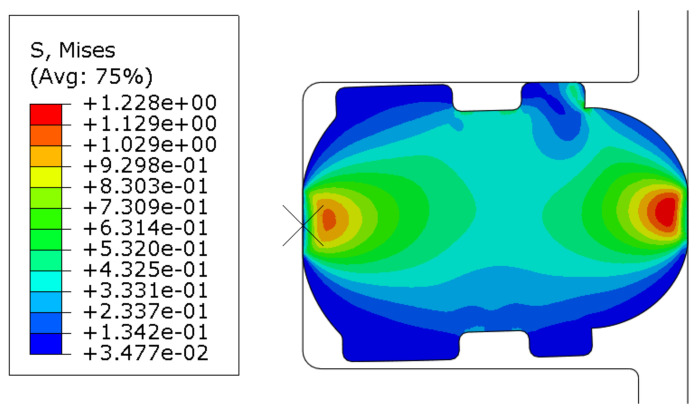
Contact deformation of sealing ring under compression of 0.5 mm.

**Figure 9:**
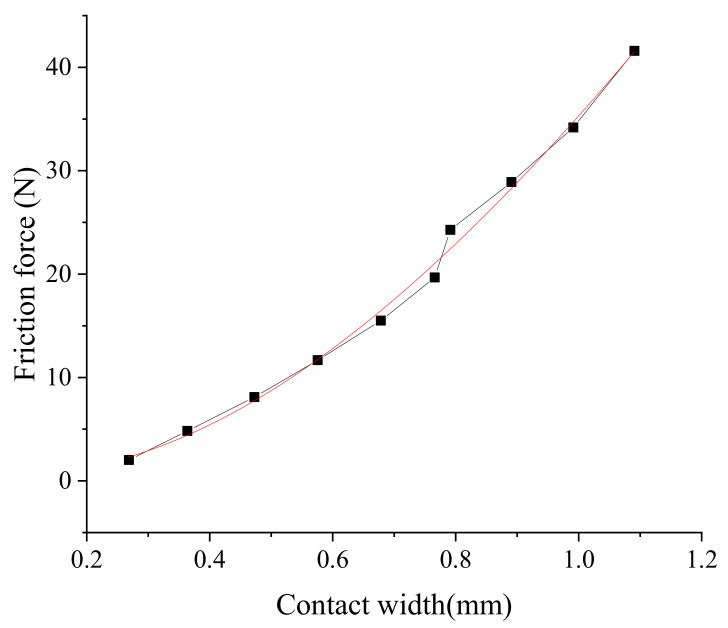
Friction under different contact widths.

The change of the contact width is mainly caused by the different compressions of the seal ring under the geometric error. Based on the finite element model, the contact width of the seal ring under different compressions can be obtained (depicted in Figure 8) and the friction force *F_Ai_* of the cylinder unit section can be calculated (depicted in Figure 9). It can be seen that the friction force increases when the contact width increases.

### 4.2. Synergy Effects on Friction Behavior

The roundness and straightness have great synergy effects on the friction behavior under the dimensional tolerance of the rubber seals and cylinder wall, such as regarding contact width (depicted in Figure 7), which changes the friction force.

**Figure 10:**
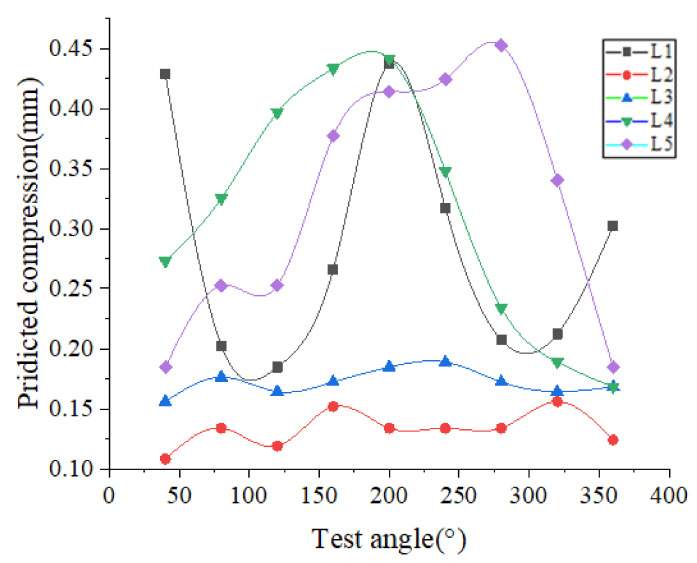
Predicted compression under synergy effects.

Here, the synergy effects of roundness and straightness on the friction behavior can be equivalent to the friction behavior under different compressions in 2D sections of rubber seals and the cylinder wall (depicted in Figure 10). Results indicate that the synergy effect on the compression is dependent on the contact area.

**Figure 11:**
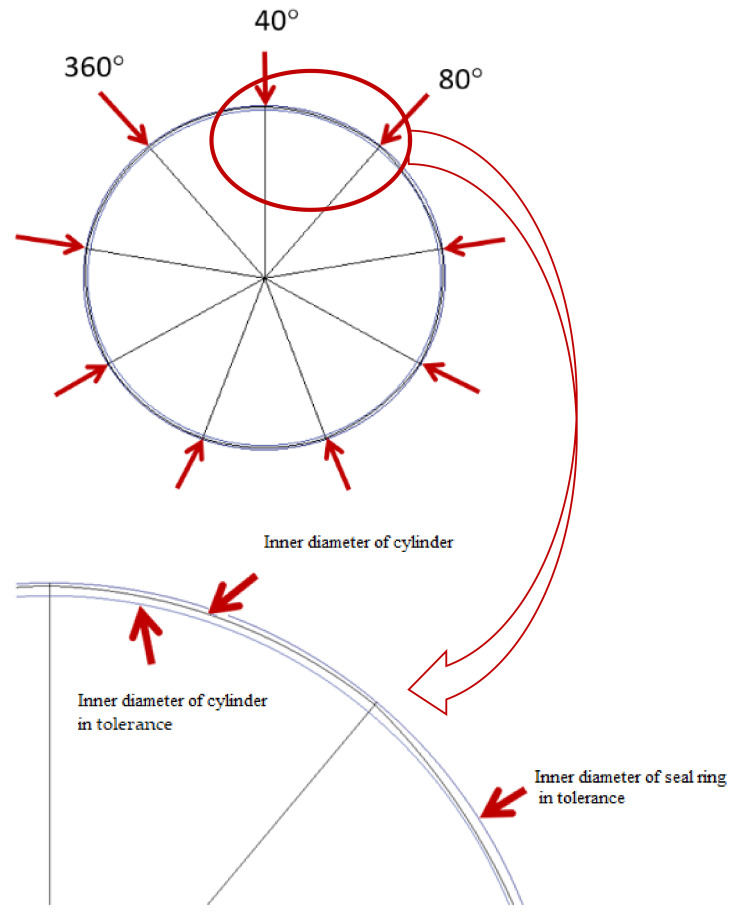
The synergy effects on compression.

At the position in L1, Figure 11 shows the synergy effects on the compression between the sealing ring and cylinder wall within the tolerance range. It is also explained that the changes in the actual seal compression lead to changes in friction behavior due to the synergy effects.

**Figure 12:**
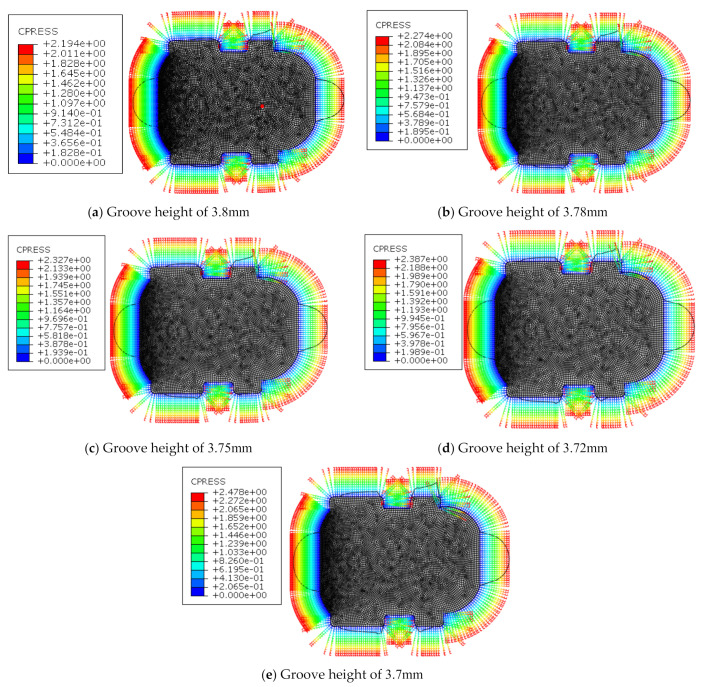
The contact pressure under different dimensions of the groove.

The dimension tolerance of the groove of the cylinder is −0.1 mm~0mm. Thus, the dimensions of the cylinder sealing ring groove were set to 3.8 mm(a), 3.78 mm(b), 3.75 mm(c), 3.72 mm(d), and 3.7mm(e) for studying the synergy effects on the friction behavior of the sealing ring. Figure 12 shows the friction analysis results of the seal ring under different seal ring groove sizes.

It can be seen that when the dimension of the cylinder seal groove decreases, the maximum contact pressure gradually increases from 2.194 MPa to 2.478 MPa. The dimension of the seal ring groove also is an important synergy factor, which has a greater influence on the contact pressure of the seal ring.

### 4.3. Friction Force

The contact widths of each angle of the cylinder were obtained in the experiment. According to the formula of the contact width and friction force obtained by the simulation, the fitting friction force of each angle can be deduced, which is denoted as *F_Ai_*. The friction force at ∆*Li* is derived from the contact width of nine points at ∆*Li*. Based on the element force to the overall force, the more points of the contact width are divided, the higher the accuracy and the more accurate the model. Then, it can be deduced that the friction force *F_Li_* of the seal at this position is given by:

(2) $$F_{Li}=\sum_{i}^{n}F_{Ai}(i=1,2,\ldots \ldots ,9{)}_{\;}$$

where *F_Li_* is the friction force at the axial ∆*Li* of the cylinder seal and *F_Ai_* is the friction force in any unit section of the cylinder.

**Figure 13:**
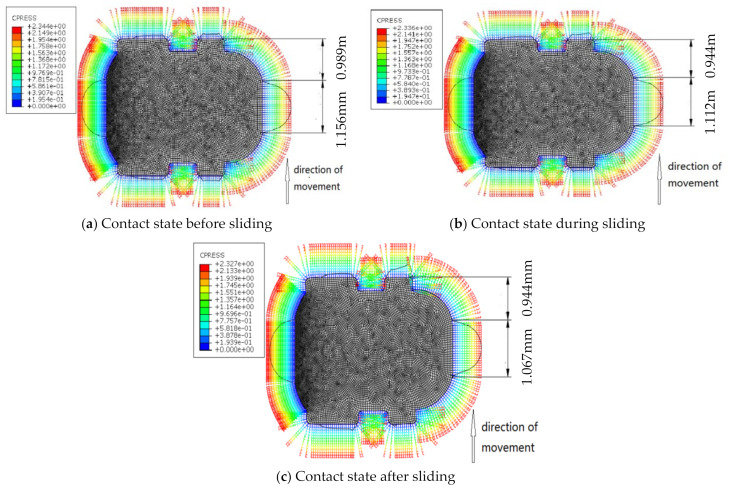
Stress diagram of seal ring simulation friction process.

Figure 13a–c respectively show the contact states of the cylinder before, during and after sliding. During the movement of the cylinder, the contact state of the seal ring surface changes (seen in Figure 13) and the position of the maximum contact pressure moves with the movement direction of the cylinder; then, it remains stable. Therefore, the dynamic friction force is characterized by the contact width during stable sliding. 

**Figure 14:**
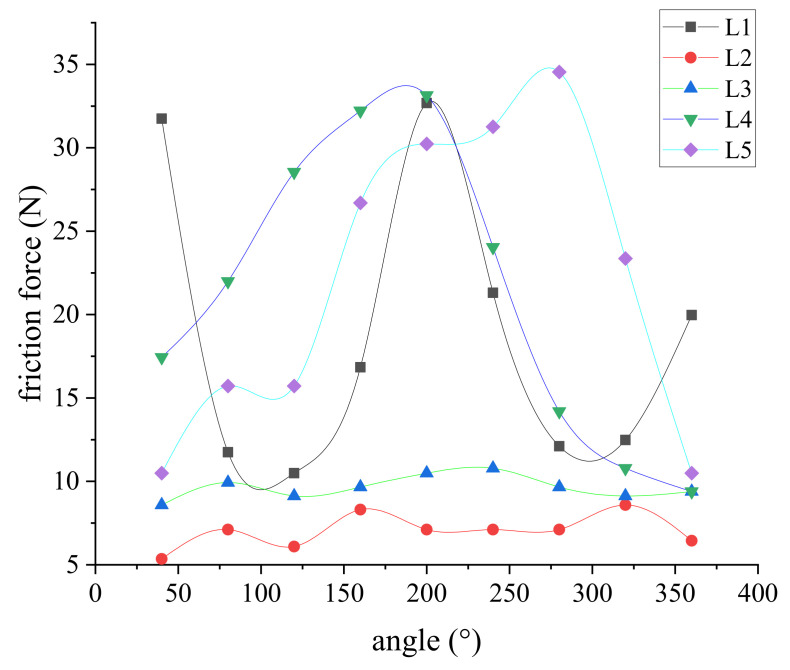
Predicted friction force in the sliding process.

Then, the contact widths at all positions of the cylinder were fitted into friction forces. Figure 14 shows the frictional forces under the angles of the cylinder, which represent the circumference variables in the test point. Results indicate that the predicted friction force was dependent on the measured results; the contact width in the sliding process was less than that in the static state of the cylinder.

**Figure 15:**
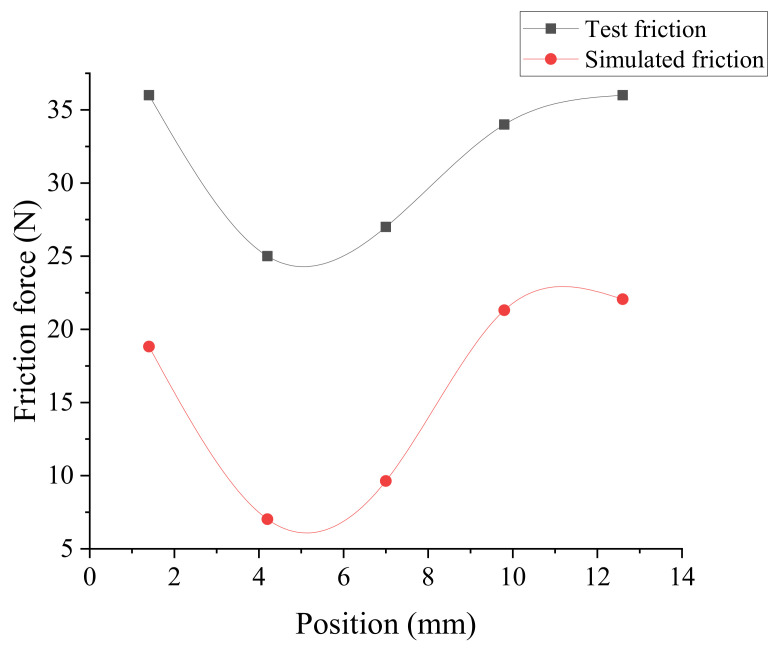
Sealing ring simulates friction and test friction.

It is assumed that the friction force is evenly distributed on the circumference. The predicted and measurement results of the friction force at the axial *Li* of the cylinder are shown in Figure 15. It can be seen that the predicted friction force is dependent on the experiment results.

## 5. Conclusions

The machining errors, such as the roundness and straightness of the inner wall of the cylinder, have a significant effect on the friction force of rubber seals, especially the synergy effects. Thus, the friction behavior of rubber seals has been investigated by using the combined method of experiment and finite element simulation. The main conclusions are given as follows:

(1) A measurement platform for the contact width of the seal ring has been developed. Here, five test points in the axial direction and nine test points in the circumferential direction were used in measuring the contact width. Results indicate that the synergy effects of roundness and straightness have great influence on the friction force; the friction force increases when the contact width increases.
(2) A finite element model of cylinder seals in the friction process has been developed for studying friction behavior. Results indicate that the predicted friction force is dependent on the measured results; the contact width in the sliding process is less than that in the static state of the cylinder.
(3) The friction behavior of rubber seals considering geometric errors has been revealed by the combined method of experiment and finite element simulation, which are useful methods for the design of cylinder geometry precision and seal structure.

## Figures and Tables

**Table 1:** The constitutive model parameters of nitrile rubber at room temperature.

*C*_10_	*C*_20_	*C*_30_
2.0028	−0.3328	0.4841

## Data Availability

The data presented in this study are available on request from the corresponding author.
